# Effect of Zirconium oxide nanoparticle on serum level of testosterone and spermatogenesis in the rat: An experimental study

**DOI:** 10.18502/ijrm.v13i9.7671

**Published:** 2020-09-20

**Authors:** Hengameh Mehdikhani, Heydar Aqababa, Ladan Sadeghi

**Affiliations:** ^1^Department of Biology, Shiraz Branch, Islamic Azad University, Shiraz, Iran.; ^2^Department of Biology, Arsanjan Branch, Islamic Azad University, Arsanjan, Iran.

**Keywords:** Zirconium oxide, Nanoparticles, Spermatogenesis, Testosterone, Rat

## Abstract

**Background:**

Zirconium nanoparticles are used as health agents, pharmaceutical carriers, and in dental and orthopedic implants.

**Objective:**

This studyaimed to investigate the effects of Zirconium oxide nanoparticles on the process of spermatogenesis in rat.

**Materials and Methods:**

In this experimental study, 32 male Wistar rats (150-200 gr), with range of age 2.5 to 3 months were used and divided into four groups of eight per each. The control group received 0.5 ml of distilled water and the three experimental groups received 50, 200, and 400 ppm doses of Zirconium oxide nanoparticles solution over a 30-day period, respectively. At the end of the experiment, tissue sections were taken from the testis and stained with hematoxylin-eosin. Serum concentration of testosterone was measured by enzyme-linked immunosorbent assay.

**Results:**

In the experimental group receiving 400 ppm Zirconium oxide nanoparticles, the number of Spermatogonia cells (p ≤ 0.01), Spermatocytes (p ≤ 0.01), Spermatids (p ≤ 0.001), and sertoli and Leydig cells (p ≤ 0.05) showed a significant decrease compared to the control group. Serum testosterone concentration did not change significantly in all experimental groups receiving Zirconium oxide nanoparticles compared to the control group. Experimental group received 400 ppm Zirconium oxide nanoparticles shrinkage of seminal tubules and reduced lumen space compared to control group.

**Conclusion:**

Zirconium oxide nanoparticles are likely to damage the testes by increasing Reactive oxygen species production and free radicals.

## 1. Introduction

Nanotechnology refers to the technology of materials that measure about 1-100 nanometers (1). The nanoparticles have unique physical and chemical properties that cannot be seen even in the materials from which they are derived. The small size and high surface area of the nanoparticles increase their chemical activity and allow them to act as a high performance catalyst (2).

Since 1960, Zirconium nanoparticles have been introduced as biomaterials for use in orthopedics in the form of artificial joints and in dentistry as coatings, implants, and composites (3). Zirconium oxide ceramic nanostructures have also been widely used in drug transducers as drug carriers for several drugs such as Myconazol, Pennyceline, Alendronit, and Zolendronitthat (4, 5). Zirconium nanoparticles have been shown to cause significant DNA damage (6) and mesenchymal stem cell death (7) and inhibit cell proliferation in rodent fibroblast cell lineages (8).

While a study by Demir and co-worker showed that Zirconium oxide nanoparticles lead to gene toxicity in peripheral blood lymphocytes and cultured renal embryonic cells (9), a study by Ye and co-worker found that high concentrations of Zirconium oxide nanoparticles induce cell death and morphological changes in cultured 3T3-E1 cells. In addition, zirconium oxide nanoparticles induced osteogenic differentiation (10). A study by Gad and co-worker showed that Zirconium oxide nanoparticles have antifungal activity against *Candida albicans* (11).

Moreover, in a study by Banerjee and co-worker, it was found that Zirconium oxide nanoparticles have antimicrobial activity (12), and Atalay and co-worker in 2018 reported that Zirconium oxide nanoparticles induce DNA damage and cell death in L929 mouse fibroblast cell liners (13). While Arefian and co-worker reported in their study that Zirconium oxide nanoparticles had strong toxic effects on liver and kidney factors (14) Omidi and co-worker showed that zirconium oxide nanoparticles at a dose of 400 ppm resulted in a significant decrease in Luteinizing hormone (LH), Follicle-stimulating hormone (FSH), and testosterone in female rats (15).

Recent reports indicate that many nanoparticles have harmful or toxic effects on spermatogenesis. In contrast, some nanoparticles have a non-toxic effect on spermatogenesis. These reports indicate that animal species, drug use, nanoparticle dosage and its characteristics including size, shape, chemical composition, surface area, and surface charge play an important role in determining the effect of nanoparticles on spermatogenesis. How nanoparticles penetrate into the blood-testicular barrier plays a crucial role in explaining the toxicity of nanoparticles on spermatogenesis (16).

According to our best knowledge, there is no study that investigate the effect of Zirconium oxide nanoparticles on spermatogenesis and serum levels of testosterone in rats. Similarly, there has been no study to evaluate the effect of Zirconium oxide nanoparticles on testicular germ cells and seminal tubular epithelium in rats for 30 days. Due to the role of Zirconium oxide nanoparticles in the production and growth of free radicals, it is likely that these nanoparticles may have pathological effects on the structure of seminal vesicles and consequently decrease sperm quality and fertility. In this study, we investigated the possible effect of Zirconium oxide nanoparticles prepared by the *Tecnan* company manufactured in Spain (CAS#1314-23-4) on serum levels of testosterone and spermatogenesis in adult rat.

## 2. Materials and Methods

### Animals

In this experimental study, 32 adult (2.5-3 month) male Wistar rats weighing approximately 150-200 gr were used. Animals were kept under the same conditions at temperatures of 20-22°C for 12 hr light/dark cycle. The animals were provided with adequate water and food and ethical considerations were observed.

### Animal treatment

The animals were divided into four groups of eight animals. Control group: received 0.5 ml of sterile distilled water. Experimental group (I): received 50 ppm Zrconium oxide nanoparticlessolution.

Experimental group (II): received 200 ppm Zirconium oxide nanoparticles solution. Experimental group (III): received 400 ppm Zirconium oxide nanoparticles solution. All solutions were given daily for 30 days orally (15). Animals body weight was measured using digital scales at the end of the experiment and were anesthetized with ether.

Blood samples were taken from the left ventricle of the heart, and the samples were kept in laboratory conditions for 20 min and centrifuged at 3000 rpm for 10 min. Then, the serum of each tube was collected. Serum concentrations of testosterone were determined by enzyme-linked immunosorbent assay.

### Histological tests

After the animals were sacrificed, their left testicles were removed. At the fixation stage, the tissues were fixed in 10% buffered formalin. The dehydration step was performed with the help of alcohol at different concentrations (from low to high). The clearing step was performed by placing the tissues in two containers containing xylene. In the replacement phase, tissues were placed in three containers of molten paraffin (65°C) for 1 hr. In the molding phase, the parts of Salto Kohart were used. At the cross-section, tissue sections were cut to 4-5 μm in thickness and hematoxylin-eosin dye for staining. All tissue studies were performed under the supervision of a pathologist. Tissue sections were morphometrically examined and imaged by a Dino-Eye-AM 423 camera. In each slide, four points were randomly selected and the walls of seminiferous tubules and the diameter of seminiferous tubules were morphometrically measured. At least five seminiferous tubules were selected from each slide. Numbers of spermatogonia, spermatocytes, spermatids, Sertoli and Leydig cells present in lumen were compared between different groups (17).

**Figure 1:**
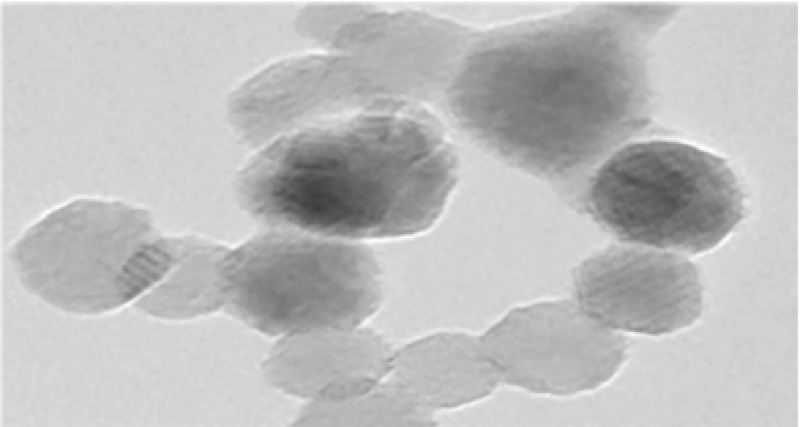
Transmission Electron Microscopy (TEM) micrograph of nanoscale zirconium dioxide suspended in media and dispersed by an ultrasonic bath (19).

**Figure 2:**
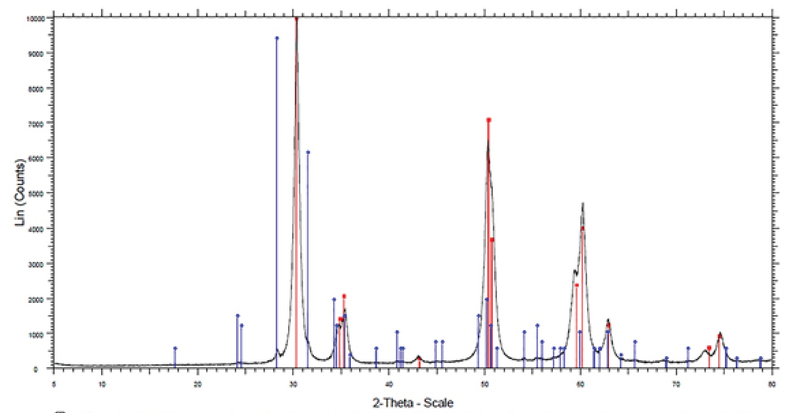
Diffractogram X-ray powder diffraction (XRD) of nanoparticles of zirconium oxide used in this experiment. The horizontal axis on theta -scale show angle between X radiancy and reflect. The vertical axis shows intensity of radiancy (19).

In this study, 14 nm zirconium nanoparticles (Figure 1) were used which were determined by X-ray diffraction methods and Transmission Electron Microscopy (TEM) and determination of extraction impurities by ICP-MS technique was 99/993% (Figure 2) (18, 19).

### Ethical consideration

All experiments were conducted according to the protocol provided by the ethics committee of Islamic Azad University Arsanjan Branch (IR.IAU.A.REC.1397.001).

### Statistical analysis

Data were analyzed using the Statistical Package for the Social Sciences (SPSS software, Chicago, Illinois), version 18.0., analysis of variance (ANOVA) and Tukey Test. Statistical inference boundary was statistically significant for the experimental groups receiving different amounts of zirconium oxide nanoparticles at P ≤ 0.05 level compared to the control group. In this study, the results of experiments performed along with the relevant statistical calculations are presented in the form of graphs.

## 3. Results

**Figure 3:**
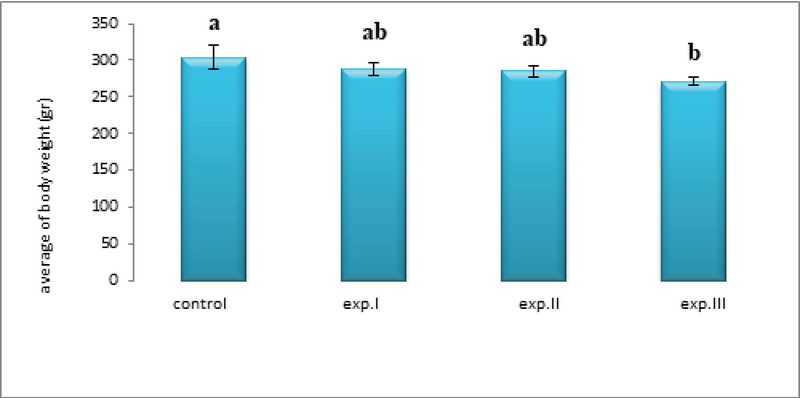
Comparison of the mean body weight in different groups. The presence of at least one similar letter is indicative of no significant difference between the groups. Lack of similar letter between the groups indicates significant difference at the level of P ≤ 0.05. Values are shown as Mean ± SEM.

While the mean body weights in the experimental groups (I) and (II) did not change significantly compared to control group, the mean body weight in the experimental group (III) significantly decreased compared to the control group (p ≤ 0.05, Figure 3).

**Figure 4:**
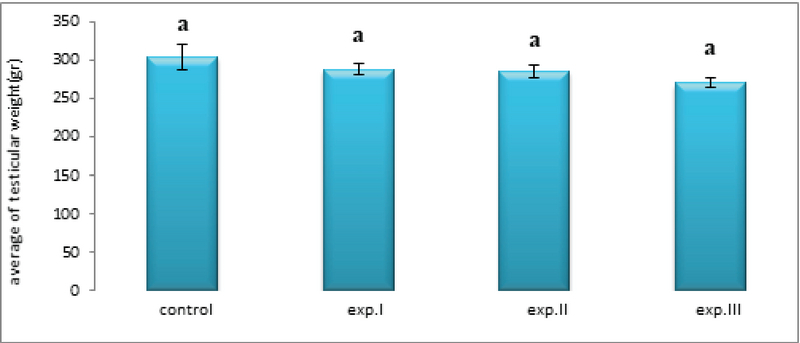
Comparison of the mean testicular weight in different groups. The presence of at least one similar letter is indicative of no significant difference between the groups. Lack of similar letter between the groups indicates significant difference at the level of P ≤ 0.05. Values are shown as Mean ± SEM.

The mean testicular weight in all experimental groups receiving different amounts of zirconium oxide nanoparticles did not change significantly compared to the control group (Figure 4).

**Figure 5:**
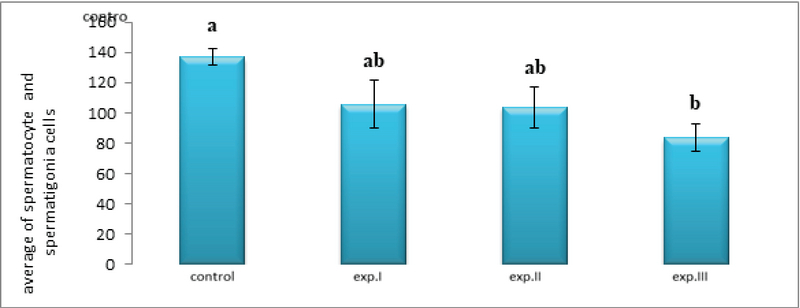
Comparison of the mean spermatocyte and spermatigonia cells count in different groups. The presence of at least one similar letter is indicative of no significant difference between the groups. Lack of similar letter between the groups indicates significant difference at the level of P ≤ 0.05. Values are shown as Mean ± SEM.

Interestingly, while the average number of spermatogonial cells in the experimental groups (I) and (II) did not change significantly compared to the control group, the average number of spermatogonial cells in the experimental group (III) decreased significantly compared to the control group (p ≤ 0.01) (Figure 5).

Further, although the average number of spermatocyte cells in the experimental groups (I) and (II) did not change significantly compared to the control group, the average number of spermatocyte cells in the experimental group (III) decreased significantly compared to the control group (p ≤ 0.01) (Figure 5).

**Figure 6:**
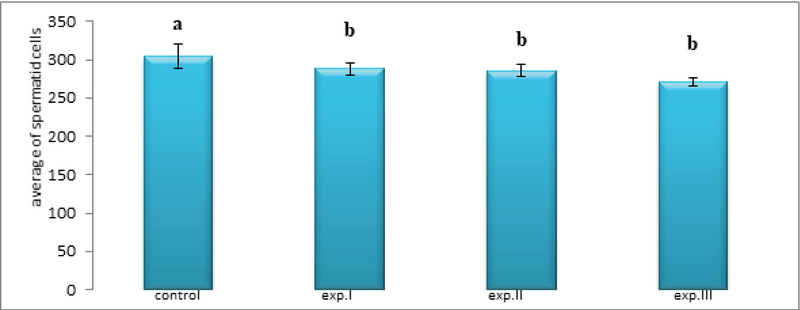
Comparison of the mean spermatid cells count in different groups. The presence of at least one similar letter is indicative of no significant difference between the groups. Lack of similar letter between the groups indicates significant difference at the level of P ≤ 0.05. Values are shown as Mean ± SEM.

However, the average number of spermatid cells in all experimental groups (I), (II) and (III) significantly decreased compared to the control group (p ≤ 0.0001, respectively) (Figure 6).

**Figure 7:**
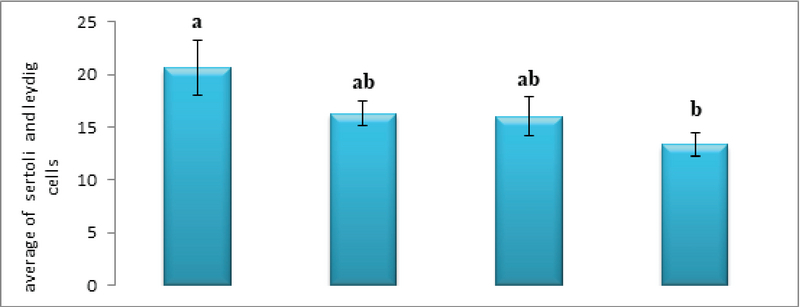
Comparison of the mean Sertoli and leydig cells count in different groups. The presence of at least one similar letter is indicative of no significant difference between the groups. Lack of similar letter between the groups indicates significant difference at the level of P ≤ 0.05. Values are shown as Mean ± SEM.

As for the Leydig cells, the average number of Leydig cells in the experimental groups (I) and (II) did not change significantly compared to the control group. However, the average number of Leydig cells in experimental group (III) decreased significantly compared to the control group (p ≤ 0.05, Figure 7).

Further, the average number of Sertoli cells in the experimental groups (I) and (II) also did not change significantly compared to the control group. However, the average number of Sertoli cells in the experimental group (III) decreased significantly compared to the control group (p ≤ 0.05, Figure 7).

**Figure 8:**
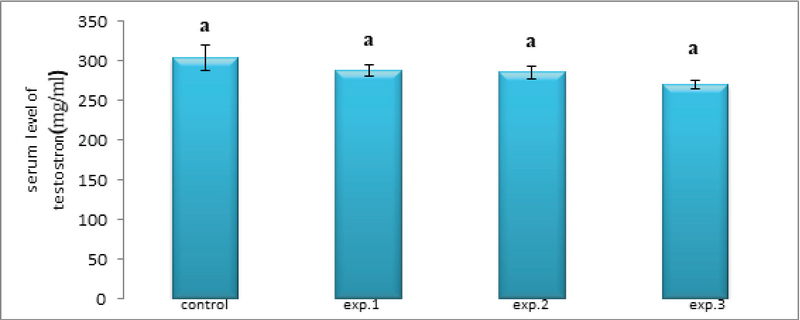
Comparison of the mean testosterone concentration in different groups. The presence of at least one similar letter is indicative of no significant difference between the groups. Lack of similar letter between the groups indicates significant difference at the level of P ≤ 0.05. Values are shown as Mean ± SEM.

The mean serum testosterone concentration in all experimental groups receiving different amounts of zirconium oxide nanoparticles did not change significantly compared to the control group (Figure 8).

### Results of testicular tissue studies

**Figure 9:**
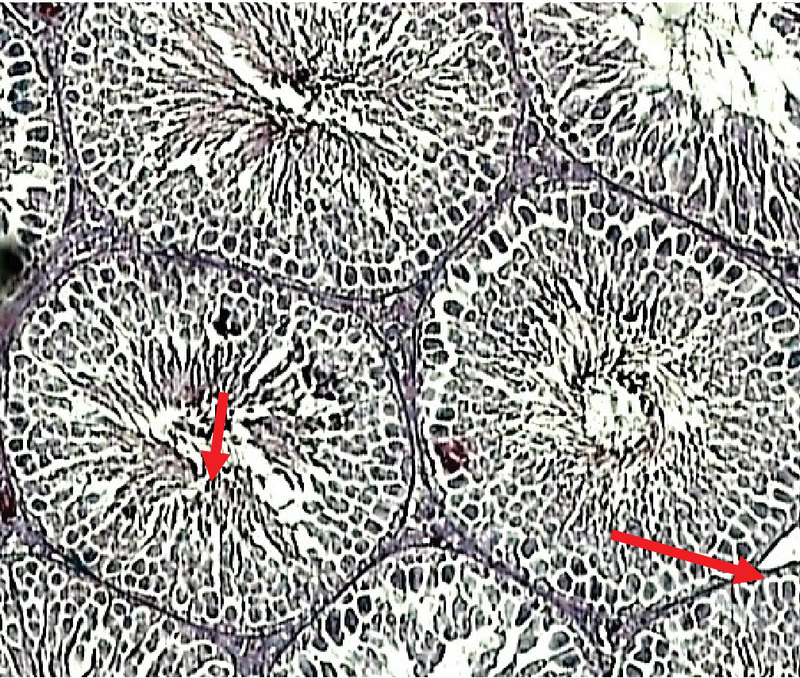
Photomicrograph of the testes in the control group. Staining: Hematoxylin and eosin (H & E), magnification '100.

There were no pathological changes in testicular photomicrographs of the control group and testicular tubules, and germ cells appeared normal (Figure 9).

**Figure 10:**
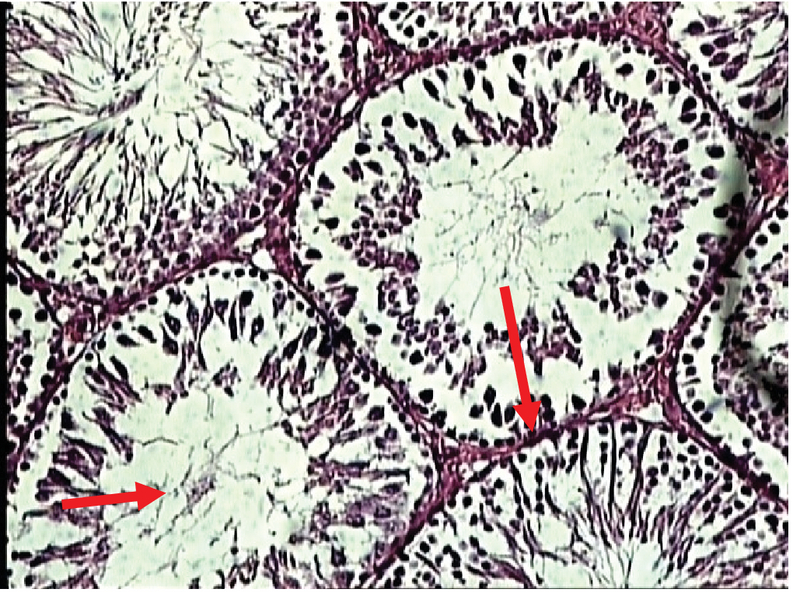
Photomicrograph of the testes in the experimental group (I) (the experimental group received 50 ppm of zirconium oxide nanoparticles). Staining: H & E, magnification '100.

In the experimental group (I), recipients of zirconium oxide nanoparticles (50 ppm) showed a decrease in the density of different types of germ cells compared to the control group (Figure 10).

**Figure 11:**
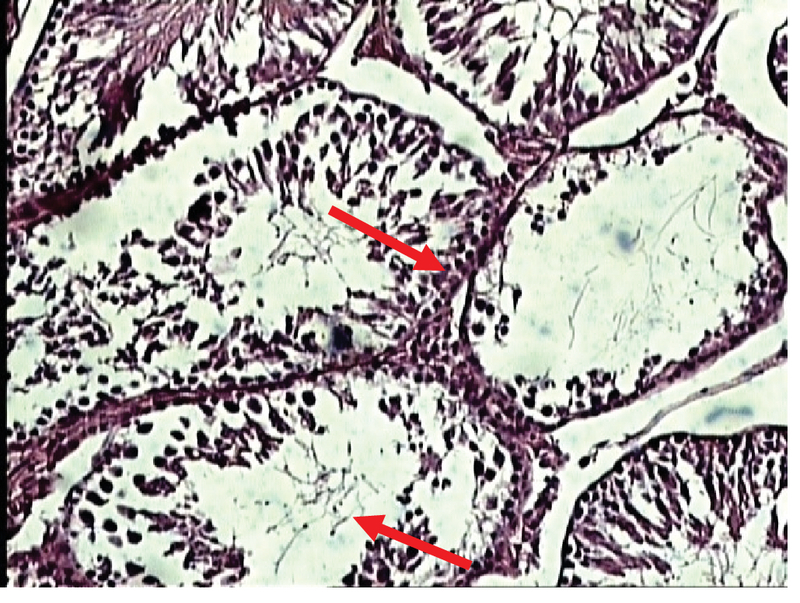
Photomicrograph of the testes in the experimental group (II) (the experimental group received 200 ppm of zirconium oxide nanoparticles). Staining: H & E, magnification ×100.

In the experimental group (II), recipients of zirconium oxide nanoparticles (200 ppm) showed better shrinkage of seminiferous tubules and decreased number of germ cells, especially spermatid cells, compared to the control group (Figure 11).

**Figure 12:**
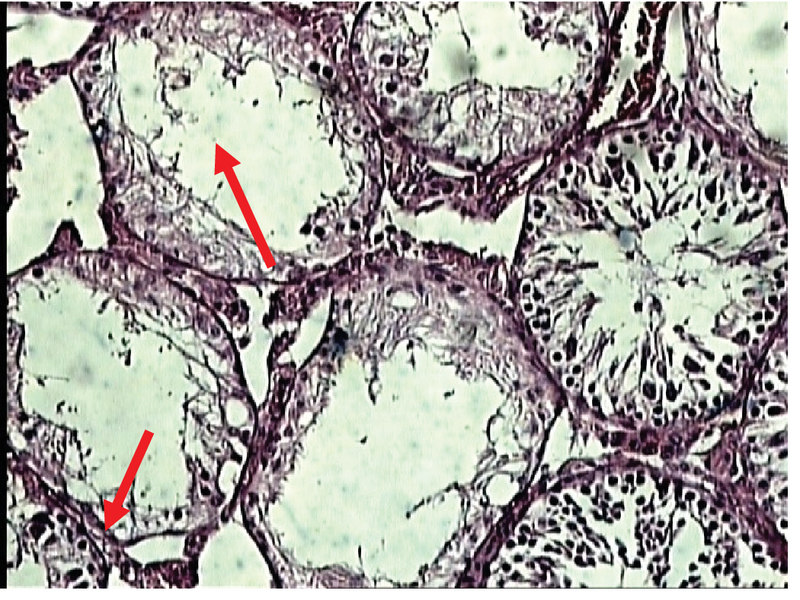
Photomicrograph of the testes in the experimental group (III) (the experimental group received 400 ppm of zirconium oxide nanoparticles). Staining: H & E, magnification ×100.

In the experimental group (III) showed a sharp decrease in the number of seminiferous tubules. The irregularity and disruption of the germinal epithelium and shrinkage of the tubules were visible. In addition, Spermatogonia, Spermatocytes, Spermatids, Sertoli and Leydig cells were noticeable in the lumen. Therefore, tissue degradation is clearly observed in this experimental group (Figure 12).

In the experimental groups receiving different amounts of Zirconium oxide nanoparticles, the number of spermatogonia, primary spermatocytes, and spermatids decreased compared to the control group. These effects are dose dependent and in the receiving group the maximum amount of Zirconium oxide nanoparticles (400 ppm) is higher than the other experimental groups.

## 4. Discussion

The mean body weight in the experimental group receiving 400 ppm zirconium oxide nanoparticles decreased significantly compared to the control group (p ≤ 0.05). These results are in line with a 2010 study by Kim and co-worker, where it was shown that the administration of 500 mg/kg of silver nanoparticles orally for 13 weeks in male mice resulted in lower body weight and increased blood cholesterol levels (20).

The mean testicular weight in all experimental groups receiving different amounts of zirconium oxide nanoparticles did not change significantly compared to the control group. A study by Fan and co-worker showed that exposure to mice with silicon oxide nanoparticles resulted in a significant decrease in testicular weight. Silicon oxide nanoparticles are likely to reduce the testicular weight of rat by oxidative damage to the testes by increasing ROS (reactive oxygen species) (21).

The average number of Sertoli cells in the experimental group receiving 400 ppm zirconium oxide nanoparticles decreased significantly compared to the control group (p ≤ 0.05). A 2013 study by Talebi and co-worker showed that zinc oxide nanoparticles damage Sertoli cells. The mechanism of the effects of nanoparticles on the testes of mice is not known. Zinc oxide nanoparticles are likely to have deleterious effects on Sertoli cell function through the formation of multinuclear giant cells, increasing vacuoles in Sertoli cells. The results of this study were consistent with the results of the study (22). Also, Braydich-Stolle and co-worker showed that silver nanoparticles decrease ATP levels in Sertoli cells by reducing FYN/Kinase enzyme levels in Sertoli cells. Silver nanoparticles appear to damage the Sertoli cells and the germ cells by reducing the amount of FYN/Kinase enzyme followed by ATP depletion. Silver nanoparticles can disrupt Sertoli cell damage by inhibiting Glial cell-derived neurotrophic factor (GDNF) signaling pathway, which is secreted from Sertoli cells, resulting in a proliferation of spermatogonial cells (23).

Further, the average number of Leydig cells in the experimental group receiving 400 ppm zirconium oxide nanoparticles decreased significantly compared to the control group (p ≤ 0.05). These results are consistent with the study of Talebi and co-worker (22). The results in their study showed that silver nanoparticles can affect Leydig cells and testosterone inhibitor synapses, and the number of Leydig cells was significantly reduced in the groups treated with silver nanoparticles (20).

Omidi and co-worker investigated the effect of zirconium nanoparticles on testosterone, LH, and FSH in female rat. Zirconium nanoparticles led to a significant decrease in plasma levels of testosterone in female rat (15). Testosterone is higher in the male than in the female. In a recent study, the dose of zirconium oxide nanoparticles used was not sufficient to influence testosterone levels in male rats. Therefore, in all groups the level of testosterone decreased but this decrease was not significant (p ≤ 0.05).

Jia and co-worker reported that titanium dioxide nanoparticles significantly reduced serum testosterone levels and expression in rat testes. Titanium dioxide nanoparticles decreased the expression of 17 beta-hydroxy steroids dehydrogenase and P 450 17 alpha-hydroxysteroid dehydrogenase and increased the expression of cytochrome P 450-19 (key enzyme for translating testosterone to estradiol) in mice. Titanium dioxide nanoparticles at the translational level cause changes in testosterone. In addition, titanium dioxide nanoparticles may reduce spermatogenesis by reducing serum testosterone synthesis (24). Due to similarities in zirconium and titanium nanoparticles in their properties, a decrease in serum testosterone level was observed in male rats treated with zirconium nanoparticle oxide, but this decrease was not significant.

Other researchers have shown that nanoparticles lead to the decline of germ cells. Miresmaeili *et al.* reported that silver nanoparticles led to a significant decrease in the number of spermatogonia, primary spermatocytes, spermatids, and spermatozoa (25).

Lan and Yang showed that nanoparticles are capable of crossing the blood-testicular barrier, thereby exerting inflammatory effects on testicular tissue. The nanoparticles do not directly destroy the nucleus membrane. Nanoparticles first affect the cytoskeleton. They then destroy the nucleus membrane. In addition, the nanoparticles may be endocytosed by sex cells or Leydig cells, causing changes in inflammation in the cell and then exocytosis (16).

Cellular and tissue damage can be caused by the extensive production of ROS in inflammatory diseases (26). In addition, increasing ROS production can lead to increased production of external cytokines and further damage (27).

Various studies have shown that zirconium nanoparticles can lead to increased production of ROS and increased caspase-3, mitochondrial membrane potential, and DNA strand breaks. Zirconium nanoparticles can cause cellular toxicity by causing mitochondrial damage (28, 13).

Studies have shown that exposure to zirconium oxide nanoparticles resulted in a significant decrease in Glutathione peroxidase (GPx) in rat. In fact, the imbalance between the production of free radicals and their ability to detoxify by GPx in the molecular mechanism of toxicity causes cell damage (29, 30, 31). Concerning the results, it seems that irconium oxide nanoparticles decrease spermatogenesis through the mechanisms described. It is also possible that zirconium oxide nanoparticles may decrease spermatogenesis by reducing serum testosterone synthesis.

## 5. Conclusion

Studies have shown that zirconium oxide nanoparticles can damage testicular tissue, which is dose-dependent. In addition, the doses of zirconium oxide nanoparticles in this study did not lead to a significant change in the serum concentration of testosterone.

##  Conflicts of Interest

The authors declare that there is no conflict of interest.
